# *Candidatus* Methanogranum caenicola: a Novel Methanogen from the Anaerobic Digested Sludge, and Proposal of *Methanomassiliicoccaceae* fam. nov. and *Methanomassiliicoccales* ord. nov., for a Methanogenic Lineage of the Class *Thermoplasmata*

**DOI:** 10.1264/jsme2.ME12189

**Published:** 2013-03-23

**Authors:** Takao Iino, Hideyuki Tamaki, Satoshi Tamazawa, Yoshiyuki Ueno, Moriya Ohkuma, Ken-ichiro Suzuki, Yasuo Igarashi, Shin Haruta

**Affiliations:** 1 Japan Collection of Microorganisms, RIKEN BioResource Center, 3–1–1 Koyadai, Tsukuba, Ibaraki 305–0074, Japan; 2 Bioproduction Research Institute, National Institute of Advanced Industrial Science and Technology (AIST), Central 6, 1–1–1 Higashi, Tsukuba, Ibaraki 305–8566, Japan; 3 Graduate School of Life and Environmental Sciences, University of Tsukuba, 1–1–1 Ten-noudai, Tsukuba, Ibaraki 305–8572, Japan; 4 Kajima Technical Research Institute, 2–19–1 Tobitakyu, Chofu, Tokyo 182–0036, Japan; 5 NITE Biological Resource Center (NBRC), National Institute of Technology and Evaluation (NITE), 2–5–8 Kazusakamatari, Kisarazu, Chiba 292–0818, Japan; 6 Department of Biotechnology, Graduate School of Agricultural and Life Sciences, The University of Tokyo, 1–1–1 Yayoi, Bunkyo-ku, Tokyo 113–8657, Japan; 7 Graduate School of Science and Engineering, Tokyo Metropolitan University, 1–1 Minami-Osawa, Hachioji-shi, Tokyo 192–0397, Japan

**Keywords:** Methanogranum caenicola, methanogen, *Thermoplasmata*, rice cluster III, anaerobic digested sludge

## Abstract

The class *Thermoplasmata* harbors huge uncultured archaeal lineages at the order level, so-called Groups E2 and E3. A novel archaeon Kjm51a affiliated with Group E2 was enriched from anaerobic sludge in the present study. Clone library analysis of the archaeal 16S rRNA and *mcrA* genes confirmed a unique archaeal population in the enrichment culture. The 16S rRNA gene-based phylogeny revealed that the enriched archaeon Kjm51a formed a distinct cluster within Group E2 in the class *Thermoplasmata* together with *Methanomassiliicoccus luminyensis* B10^T^ and environmental clone sequences derived from anaerobic digesters, bovine rumen, and landfill leachate. Archaeon Kjm51a showed 87.7% 16S rRNA gene sequence identity to the closest cultured species, *M. luminyensis* B10^T^, indicating that archaeon Kjm51a might be phylogenetically novel at least at the genus level. In fluorescence *in situ* hybridization analysis, archaeon Kjm51a was observed as coccoid cells completely corresponding to the archaeal cells detected, although bacterial rod cells still coexisted. The growth of archaeon Kjm51a was dependent on the presence of methanol and yeast extract, and hydrogen and methane were produced in the enrichment culture. The addition of 2-bromo ethanesulfonate to the enrichment culture completely inhibited methane production and increased hydrogen concentration, which suggested that archaeon Kjm51a is a methanol-reducing hydrogenotrophic methanogen. Taken together, we propose the provisional taxonomic assignment, named *Candidatus* Methanogranum caenicola, for the enriched archaeon Kjm51a belonging to Group E2. We also propose to place the methanogenic lineage of the class *Thermoplasmata* in a novel order, *Methanomassiliicoccales* ord. nov.

Until recently, the class *Thermoplasmata* had consisted of mainly acidophilic, aerobic, mesophilic to thermophilic, and sulfur-reducing archaea such as genera *Acidiplasma* (15), *Ferroplasma* (14), *Picrophilus* (47), *Thermoplasma* (4), *Thermogymnomonas* (28), and *Candidatus* Acidulipro-fundum boonei (50). Archaeal members of those genera mainly inhabit extreme environments such as acidic and solfataric fields. On the other hand, culture-independent approaches have retrieved a diverse array of environmental clones belonging to the class *Thermoplasmata* from ordinary environments, and many of these clones form huge uncultured archaeal lineages at the order level, so-called Groups E2 and E3 (6, 33, 39). Groups E2 and E3 consist of sublineages such as Marine group II, deep-sea hydrothermal vent Euryarchaeotic group 1 and 2 (DHVE1 and DHVE2), and rice cluster III (RC-III), which is derived from the alimentary canal (12, 19, 54), anaerobic digester (13), contaminated aquifer (8), deep-sea hydrothermal vent (40, 55), marine plankton (5, 7), and rice field soil (3, 17, 30). More recently, a uniformly shaped pure culture B10^T^, given the name *Methanomassiliicoccus luminyensis*, was isolated from human feces, and revealed to be a methanol-reducing, mesophilic, slightly alkaliphilic methanogen belonging to the class *Thermoplasmata* (9). These findings suggest that the class *Thermoplasmata* is a phenotypically versatile taxon; however, very little is known about the phylogenetic diversity and ecological distribution of methanogens in the class *Thermoplasmata*.

In our previous study, members of RC-III within Group E2 in the *Thermoplasmata* as well as *Methanoculleus*, *Methanosarcina*, and *Methanothermobacter* species have been detected from methanogenic bioreactors (1, 21, 43, 44, 46). To obtain cultures of those methanogens, we conducted enrichment cultures from methanogenic digester sludge and eventually succeeded in enriching a novel methanogen belonging to Group E2 in class *Thermoplasmata*. Thus, this paper deals with the phylogenetic characterization of the enriched methanogen in *Thermoplasmata* and the provisional characterization of the phenotypes.

## Materials and Methods

### Sampling

The anaerobic sludge was collected from a methanogenic packed-bed reactor at Kajima Technical Research Institute on 16th December 2004. The reactor, which was packed with carbon fiber textile as supporting media (43–45), had been properly operated at 55°C and was stably producing methane gas from garbage slurry as feedstock. The garbage slurry was prepared from kitchen waste from the company cafeteria. It was diluted with an equal amount of water after removing non-biodegradable materials and then pulverized using a homogenizer. The physicochemical properties of the slurry were as follows: pH 5.2; chemical oxygen demand (COD), approx. 203 g L^−1^; and volatile suspended solids (VSS), approx. 104 g L^−1^.

### Enrichment from the sludge

The basal medium was used with or without 0.01% (w/v) yeast extract (Becton Dickinson, Franklin Lakes, NJ, USA), designated YB and B media, respectively, in this study. Basal medium was composed of (L^−1^): 0.54 g NH_4_Cl, 0.14 g KH_2_PO_4_, 0.20 g MgCl_2_·6H_2_O, 0.15 g CaCl_2_·2H_2_O, 2.5 g NaHCO_3_, and 1.0 mL trace element solution (58) containing 4.0 mg Na_2_WO_4_·H_2_O and eliminating NaCl. Prior to inoculation, the pH of the medium was adjusted to 7.0 with 6 N HCl, dissolved oxygen was removed by flushing with N_2_:CO_2_ (4:1, v/v), and 10 mL vitamin solution (L^−1^) (60) and 10 mL sterile stock solution of Na_2_S/cysteine-HCl solution (each 50.0 g L^−1^) (26) were added. H_2_:CO_2_ (4:1, v/v; approx. 150 kPa), formate, acetate, or methanol (all at 10 mM) was added to the basal medium as the sole substrate. For enrichment, 0.5 mL anaerobic sludge was inoculated into 20 mL of each medium and incubated at 30°C for a week. A stable enrichment culture was obtained after three cultivations in MYB medium, YB medium supplied with methanol. The enrichment culture was maintained in MYB medium by consecutive transfer monthly.

### Preparation of DNA, PCR amplification, and DNA sequencing

The genomic DNA was extracted from the enrichment culture and purified as described previously (49). The archaeal and bacterial 16S rRNA genes were amplified by PCR using the following primers: A10F (5′-TCYGGTTGATCCYGCCRG-3′) and A1400R (5′-ACGGGCGGTGTGTGCAAG-3′) for the domain *Archaea*, U27F and U1492R (25) for the domain *Bacteria*. The PCR mixture (50 μL) contained 1×PCR buffer, 3.5 mM MgCl_2_, 10 mM deoxynucleoside triphosphates (dNTPs), 1.25 U AmpliTaq Gold (each from Applied Biosystems, Foster City, CA, USA), and 0.4 μM of each forward and reverse primer. Approximately 100 ng genomic DNA was used as a template under the following cycling conditions: initial AmpliTaq Gold activation at 95°C for 9 min, followed by 30 cycles of denaturation at 95°C for 30 s, annealing at 56°C for 30 s, extension at 72°C for 1.5 min, and a final extension step at 72°C for 5 min. The *mcrA* gene encoding the alpha-subunit of methyl-coenzyme M reductase was also partially amplified by PCR with primers MR1mod and ME2mod (35) under almost the same PCR conditions except for its cycle number (40 cycles) and time of extension step (1 min) in the cycle. The PCR product was purified using the QIAquick PCR purification kit (Qiagen, Hilden, Germany), and sequenced using the BigDye terminator v3.1 cycle sequencing kit with a 3130*xl* genetic analyzer (both from Applied Biosystems).

### Clone library

The purified archaeal 16S rRNA and *mcrA* genes were cloned with a pT7Blue T-vector kit (Novagen, Madison, WI, USA). The clonal DNAs were amplified from randomly selected recombinants by direct PCR with M13 primers, and then used as templates for sequencing. A universal primer 907r (56) and T7 promoter primers were used for sequencing the cloned 16S rRNA and *mcrA* genes, respectively. The obtained sequences of all the 16S rRNA gene clones (~690 bp) and the *mcrA* gene clones (~475 bp) were compared with those in the GenBank database using the BLAST program (NCBI-BLAST, www.ncbi.nlm.nih.gov/BLAST), and aligned using the CLUSTAL_X program. Sequence identity of 99% was used as the cut-off value for grouping the sequences into different operational taxonomic units (OTUs).

### Phylogenetic analyses

Almost full-length 16S rRNA gene and partial *mcrA* gene sequences were determined for phylogenetic analysis. The following primers were used for sequencing the PCR product of the archaeal 16S rRNA gene: A10F, Kjm700F (5′-TGGGGTAGGGGTAAA ATCCT-3′), Kjm1000F (5′-ACTCACCAGGGGAGACTGTT-3′), A500R (5′-GTGTTACCGCGGCKGCTGG-3′), Kjm700R (5′-GTG GTCCTTCTAGGATTACA-3′), and A1400R, that of the bacterial 16S rRNA gene: U520F (5′-GTGCCAGCAGCCGCGG-3′) and U1492R, and that of *mcrA* gene: MR1mod and ME2mod. Sequences were compared using the BLAST program with those available in the DDBJ/EMBL/GenBank databases. Phylogenetic analyses were carried out using the 16S rRNA gene sequence and deduced amino acid sequence of the *mcrA* gene. The 16S rRNA gene sequences were aligned with an ARB data set using ARB software (32). According to the previously described method (27), thirty-five reference sequences of the phylogenetically related archaea and environmental clones were selected as authentic sequences located in the class *Thermoplasmata*. The data set of deduced McrA amino acid sequences was aligned using the CLUSTAL_X program. Phylogenetic trees were constructed by the neighbor-joining (NJ) method with the CLUSTAL_X program (42, 57) and the maximum-likelihood (ML) method with MORPHY software version 2.3b3 (10, 20). In addition, the posterior probabilities of branching points were estimated by Bayesian inference using MrBayes 3.1 (23, 41).

### Fluorescence *in situ* hybridization

The enriched archaeon Kjm51a grew on the aforementioned MYB medium for 8 d. The harvested cells were fixed in 4% paraformaldehyde at 4°C for 2 h and stored in 99% ethanol–phosphate-buffered saline (1:1). The fixed cells were incubated in hybridization buffer (0.9 M NaCl, 0.01% sodium dodecyl sulfate, 20 mM Tris-HCl, pH 7.2, and an appropriate amount of formamide) containing fluorescently labeled probes (0.5 pmol μL^−1^). After incubation at 46°C for 10 h, the buffer was replaced with washing solution (378 mM NaCl, 0.01% sodium dodecyl sulfate, 20 mM Tris-HCl, pH 7.2, and 5 mM EDTA). The sample was incubated at 46°C for 20 min. and then stained with 1 μg mL^−1^ of 4′,6-diamidino-2-phenylindole (DAPI). The sample obtained was observed under a confocal laser scanning microscope (LSM710; Carl Zeiss Microscopy, Tokyo, Japan). A specific oligonucleotide probe targeting the 16S rRNA gene of the enriched archaeon Kjm51a (RC281r2, 5′-AAGGCCCATACCCGTCATC-3′) was designed using the Probe Design tool of the ARB software package (32). The overall Gibbs free energy of this probe and target sequence calculated with the mathFISH web server was −9.2 kcal mol^−1^ (62). The probes were labeled with fluorescent dye, Alexa Fluor 555 (Japan Bio Services, Saitama, Japan). Two domain-specific probes were also used: EUB338 labeled with Alexa Fluor 647 for detection of almost all bacteria (2), and ARC915 labeled with Alexa Fluor 488 for detection of almost all archaea (53). The stringency of hybridization was adjusted by adding formamide to the hybridization buffer (15% [v/v] for all the probes used in this study). More than 8,000 DAPI-stained cells were counted to determine the ratio of ARC915-hybridized cells to EUB338-hybridized cells.

### Physiological characteristics

Growth conditions were determined using MYB medium. Aerobic and microaerobic conditions were prepared by the substitution of air and the addition of 2% (v/v) oxygen, respectively, with filtration through a 0.2 μm-pore membrane filter. Prior to inoculation, acetate, lactate, or pyruvate (all at 10 mM) were added as carbon sources instead of yeast extract. 2-Bromo ethanesulfonate (BES, final concentration 20 mM) was added as the inhibitor of methane production. Then, 0.2 mL of the preculture of the enrichment was inoculated into 20 mL fresh medium containing each substrate. The culture was incubated at 30°C for two weeks. After the transfer twice, hydrogen and methane concentrations in the headspaces of serum bottles were determined with a gas chromatograph (GC-14A; Shimadzu, Kyoto, Japan) equipped with a thermal conductivity detector and a porapack Type Q 80–100, mesh 80–100 (Waters, Tokyo, Japan). The analysis conditions were as follows; column temperature, 60°C; injector temperature, 80°C; and detector temperature, 100°C; current, 80 mA; carrier gas, N_2_.

### Accession numbers

The 16S rRNA gene and *mcrA* gene sequences of the enriched archaeon Kjm51a have been deposited in the DDBJ/EMBL/NCBI, and GenBank nucleotide sequence databases under accession numbers AB749767 and AB749768, respectively.

## Results

### Methanogenic enrichment cultures from anaerobic sludge

A methanogenic enrichment culture was obtained from anaerobic digester using MYB medium containing methanol and yeast extract. The archaeal population in the culture was analyzed using archaeal 16S rRNA gene- and *mcrA* gene-specific primers. A total of 113 and 61 clones were obtained, respectively. A sole phylotype was obtained in both clone libraries, *i.e.*, all the cloned 16S rRNA gene and *mcrA* gene sequences in the two libraries were almost identical to the sequence identities of 99.4–100% and 99.5–100%, respectively.

**Fig. 1:**
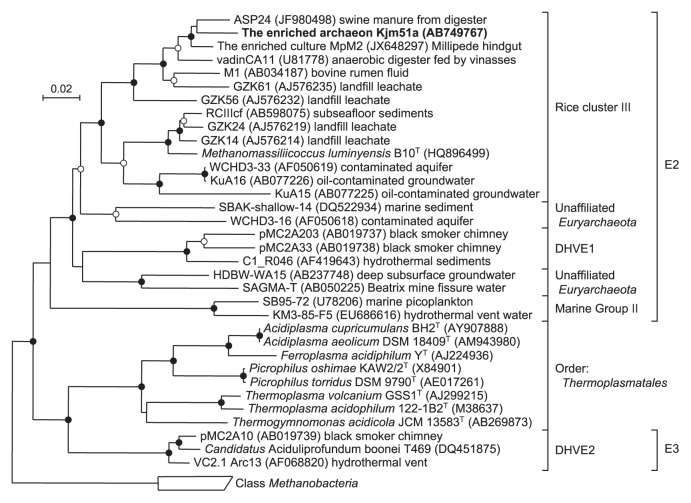
Phylogenetic affiliation of the enriched archaeon Kjm51a within Group E2 in the class *Thermoplasmata* on the basis of the 16S rRNA gene sequences. The tree was constructed using the neighbor-joining method. Solid circles at branching nodes indicate supporting probabilities above 95% by all the phylogenetic analysis methods (NJ, ML, and Bayesian), and open circles indicate probabilities above 85% by two or more analyses. Bar, 0.02 substitutions per nucleotide position.

**Fig. 2:**
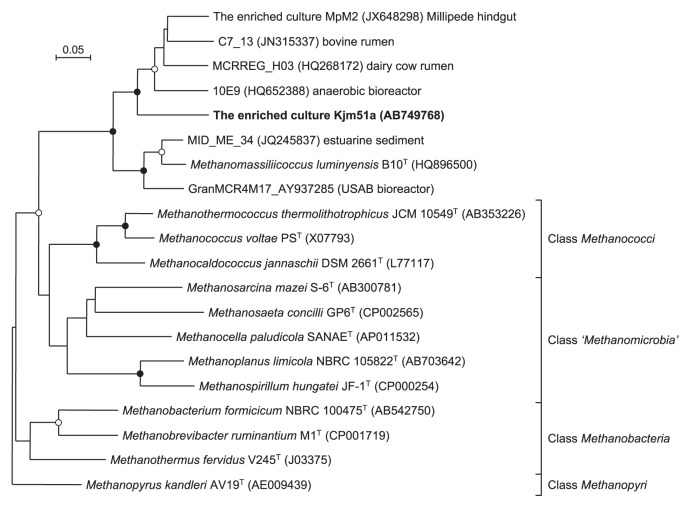
Deduced McrA amino acid sequence-based phylogeny showing the phylogenetic relationships among the enriched archaeon Kjm51a, its related archaeon and environmental clones, and other known methanogens. Solid circles at branching nodes indicate supporting probabilities above 95%, and open circles indicate probabilities above 80%. Bar, 0.02 substitutions per nucleotide position.

An almost full-length 16S rRNA gene sequence (1,309 bp) was determined for a novel archaeon, designated phylotype Kjm51a, in the enrichment culture. In the phylogenetic trees of the 16S rRNA gene sequences constructed using NJ, ML, and Bayesian methods, the enriched archaeon Kjm51a was placed into an uncultured archaeal lineage, Group E2, in the class *Thermoplasmata* (6) (Fig. 1). The topologies of the trees generated by the three phylogenetic analysis methods were almost identical, and were supported by high bootstrap values (99–100%). Archaeon Kjm51a was a member of RC-III, a sublineage within Group E2, and showed the highest sequence similarities (91.3–96.2%) to the environmental clones derived from anaerobic digesters, bovine rumen, and landfill leachate (13, 22, 54, 61). The nearest cultivated neighbor of archaeon Kjm51a was *Methanomassiliicoccus luminyensis* B10^T^ with 87.7% sequence identity. A partial *mcrA* gene sequence (1,109 bases) was also determined for the enriched archaeon Kjm51a. The NJ tree constructed using the McrA amino acid sequence deduced from the *mcrA* gene sequence demonstrated that archaeon Kjm51a formed a monophyletic cluster together with *M. luminyensis* B10^T^ and environmental clone sequences derived from anaerobic bioreactor and bovine rumen (Fig. 2), and that the cluster was apparently distinct from the four known methanogenic lineages, the classes *Methanobacteria*, ‘*Methanomicrobia*’, *Methanococci*, and *Methanopyri*. The McrA amino acid sequence of the enriched archaeon Kjm51a showed 76.0% identity with that of the closest species, *M. luminyensis* B10^T^.

**Fig. 3:**
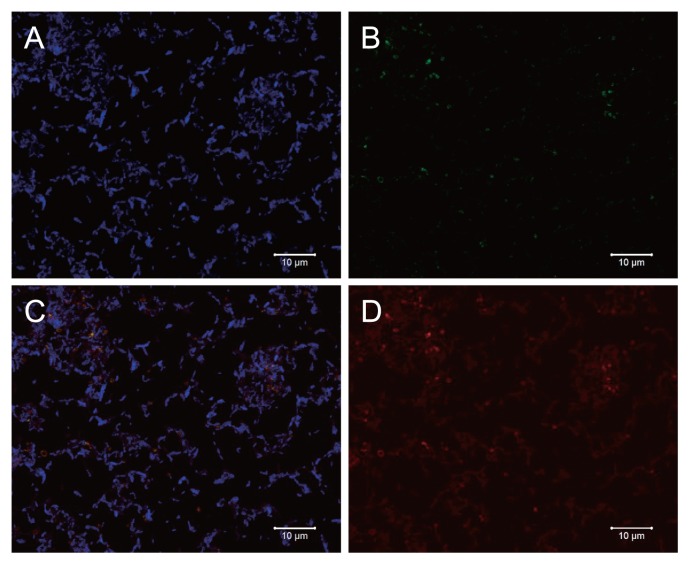
Epifluorescence micrographs of *in situ* hybridization of the enrichment archaeon Kjm51a grown on MYB medium for a week. The same microscopic field is shown after hybridization with a Kjm51a-specific probe (red), an archaeal probe ARC915 (green), and a bacterial probe EUB338 (blue). A, blue color; B, green color; C, merge of red, green and blue colors; D, red color. Bars, 10 μm.

Coccoid- and rod-shaped cells were observed under the microscope. The cocci and rods were identified as archaeal and bacterial cells, respectively, by fluorescence *in situ* hybridization with archaeal and bacterial probes (Fig. 3A and B). A ratio of archaeal cells to total cells was at least 3.5±1.4% in the enrichment culture. Cocci were hybridized with a Kjm51a-specific probe, but rods were not (Fig. 3C). Cells hybridized with the Kjm51a-specific probe completely corresponded to those with the archaeal probe (Fig. 3B and D). The bacterial rods in the enrichment culture were provisionally identified as *Clostridium celerecrescens* (sequence identity: 99.9%, X71848) by bacterial 16S rRNA gene sequence analysis.

### Physiological property of the enriched archaeon Kjm51a

**Fig. 4:**
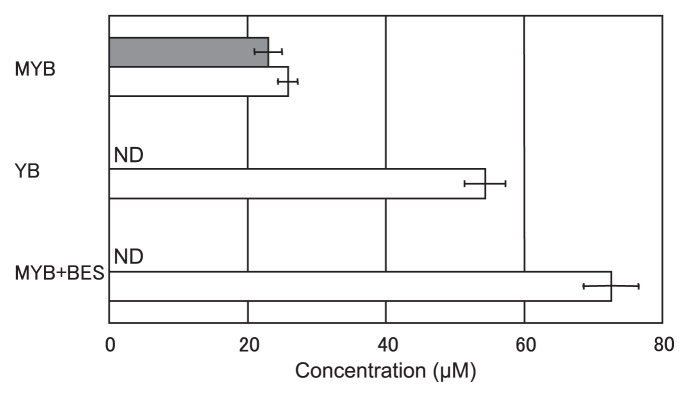
Methane and hydrogen production in the enrichment culture obtained from anaerobic sludge. Filled bars, methane; open squares, hydrogen. Data points and bars are the means and standard deviations, respectively (*n*=3). Abbreviations: MYB, MYB medium supplied with methanol in YB medium; YB, YB medium; MYB+BES, MYB medium supplied with BES; ND, not detected.

The enriched archaeon Kjm51a was strictly anaerobic and was capable of growing in MYB medium under a N_2_/CO_2_ (4:1 [v/v]) atmosphere, but could not grow under microaerobic or aerobic conditions. Both methanol and yeast extract were required for the growth of archaeon Kjm51a. Acetate, lactate, and pyruvate were not utilized as carbon sources instead of yeast extract. Metabolic products of the enrichment culture with methanol and yeast extract were hydrogen and methane, and that with yeast extract and without methanol was hydrogen (Fig. 4). Growth of the enriched archaeon Kjm51a in the presence of methanol and its methane production were completely inhibited by the addition of BES. The amount of hydrogen in the presence of BES was approximately three times higher than that in the absence of BES.

## Discussion

The novel archaeon Kjm51a was successfully enriched from anaerobic sludge using MYB medium containing methanol and yeast extract. The archaeon was not yet purified in this study because *C. celerecrescens* was dominantly isolated in the presence of yeast extract, although we made a great effort to isolate the archaeon using agar plate culture and dilution-extinction culture. Clone library analysis demonstrated that cloned 16S rRNA and *mcrA* gene sequences obtained from the enrichment culture were almost identical in each. Furthermore, FISH analysis also showed that coccal cells hybridized with a Kjm51a-specific probe completely corresponded to those with an archaeal probe. These findings strongly support the archaeal purity of the enriched archaeon Kjm51a in MYB medium, although bacterial cells still coexisted.

The enriched archaeon Kjm51a was a strictly anaerobic and chemoheterotrophic cocci showing growth and methane production in the presence of methanol, the inhibition of methane production by BES, a well-known inhibitor of methanogenesis (18). Hydrogen production was also observed in the absence of methanol, which indicated that the coexisting bacterium, *C. celerecrescens*, produced hydrogen by its fermentation (38). Inhibition of methane production by BES resulted in increased hydrogen production. These physiological properties suggest that the enriched archaeon Kjm51a might be a methanol-reducing hydrogenotrophic methanogen.

As reported for *Methanosphaera stadtmanae* (34), *Methanomicrococcus blatticola* (51), and *Methanosarcina barkeri* strain Fusaro (36), the enriched archaeon Kjm51a is likely to produce methane by the hydrogen-dependent reduction of methanol through the following reaction: H_2_+ CH_3_OH → CH_4_+H_2_O (11, 29, 52, 59). *Methanomassiliicoccus luminyensis*, a recently isolated methanogen from human feces, belonging to Group E2, also produced methane from methanol in the presence of hydrogen (9). The genome of *M. luminyensis* likely encodes only a partial methanogenesis pathway (16). Most recently, archaeon MpT1 in Group E2 was enriched from termite guts as a methanogen, which converted methanol to methane (39). Methanol may be a common substrate for methanogenesis in Group E2. Schink and Zeikus reported that heterotrophic microbes anaerobically produced methanol as a major end product from pectin, which is a component of plant tissue (48). Biodegradation of plants occurs in a wide variety of environments, such as the rumen, rice field soil and anaerobic digester treating garbage, and Group E2 methanogens may contribute to carbon flux.

The enriched archaeon Kjm51a is the first culture representative derived from an anaerobic methanogenic digester in Group E2. In the phylogenetic trees constructed using 16S rRNA gene sequences, the archaeon Kjm51a and *M. luminyensis* B10^T^ were completely separated in Group E2 with their low sequence identity (87.7%), which was sufficiently low to classify them into different genera. The enriched archaeon Kjm51a and *M. luminyensis* B10^T^ were affiliated with RC-III, one of the sublineages in Group E2. RC-III was clearly and completely separated from the validly described order *Thermoplasmatales*. Its monophyletic lineage was strongly supported by the probability scores (>99%) calculated using all the phylogenetic analysis methods. The 16S rRNA gene sequence of the enriched archaeon Kjm51a and *M. luminyensis* B10^T^ had similarities of only 77.1 to 80.3% with those of the known archaeal species in the order *Thermoplasmatales*. These similarities are lower than the 85% similarity that is generally used as a cut-off value for distinguishing lineages at the phylum, as suggested by Hugenholtz *et al.* (24). Therefore, RC-III composed of the enriched culture Kjm51a and *M. luminyensis* B10^T^ is a distinct order level lineage in the class *Thermoplasmata*. Previously, Kemnitz *et al.* (30) reported that the RC-III archaea might heterotrophically grow using peptides, based on their enrichment culture experiment; however, taken together with the recent study (6), our findings clearly indicated that RC-III is a novel methanogenic lineage.

In conclusion, an archaeal representative enriched from the anaerobic methanogenic digester is a novel methanogen belonging to RC-III within Group E2 in the class *Thermoplasmata*. According to the recommendations of Murray and Stackebrandt (37), we propose the provisional taxonomic assignment of *Candidatus* Methanogranum caenicola for the enriched archaeon Kjm51a. Most recently, the order *Methanoplasmatales* was provisionally proposed for the deep-branching lineage accommodating *M. luminyensis* and the enriched archaea MpT1 and MpM2 (39); however, this lineage should be proposed as the *Methanomassiliicoccales* on the basis of Rule 47a of the Bacteriological Code to avoid bacteriological confusion (31). Consequently, we propose to rename the order ‘*Methanoplasmatales*’ as *Methanomassiliicoccales* for the sublineage accommodating *M. luminyensis* B10^T^ and the enriched archaeon Kjm51a as described below. The proposal of this novel order follows the description of the new family *Methanomassiliicoccaceae*. To purify the enriched archaeon Kjm51a and understand its ecological role in the methanogenic environment, further study via enrichment culture will be necessary.

## Description of *Candidatus* Methanogranum caenicola

Methanogranum caenicola (Me.tha.no.gra’num. cae.ni. co’la. N.L. n. methanum [from French n. méth(yle) and chemical suffix -ane], methane; N.L. pref. methano-, pertaining to methane; L. neut. n. granum, grain, kernel; N.L. neut. n. Methanogranum, a methane-producing grain: L. n. caenum, mud, sludge; L. suff. -cola [from L. n. incola], inhabitant, dweller; N.L. n. caenicola, an inhabitant of sludge).

Strictly anaerobic, chemoheterotrophic. Cells form cocci occurring as single cells. Produce methane dependent on hydrogen and methanol. Represent a distinct phylogenetic lineage in the class *Thermoplasmata* based on 16S rRNA gene sequence analysis. Enriched from an anaerobic sludge in a methanogenic digester.

## Description of *Methanomassiliicoccaceae* fam. nov

*Methanomassiliicoccaceae* (Me.tha.no.mas.si.li.i.coc.ca’ce.ae. N.L. neut. n. *Methanomassiliicoccus* type genus of the family; *-aceae* ending to denote a family; N.L. fem. pl. n. *Methanomassiliicoccaceae* family of the genus *Methanomassiliicoccus*).

The family *Methanomassiliicoccaceae* is defined on the basis of a phylogenetic tree constructed by phylogenetic analysis of the 16S rRNA gene sequence of a single cultivated representative, of the enriched culture, and of environmental clone sequences derived mainly from the alimentary canal, anaerobic digester, landfill leachate, and rice field soil. The type genus is *Methanomassiliicoccus*.

## Description of *Methanomassiliicoccales* ord. nov

*Methanomassiliicoccales* (Me.tha.no.mas.si.li.i.coc.cal’es. N.L. neut. n. *Methanomassiliicoccus* type genus of the order; *-ales* ending to denote an order; N.L. fem. pl. n. *Methanomassiliicoccales* order of the genus *Methanomassiliicoccus*).

The description is the same as that for the family *Methanomassiliicoccaceae*. The type genus is *Methanomassiliicoccus.*
